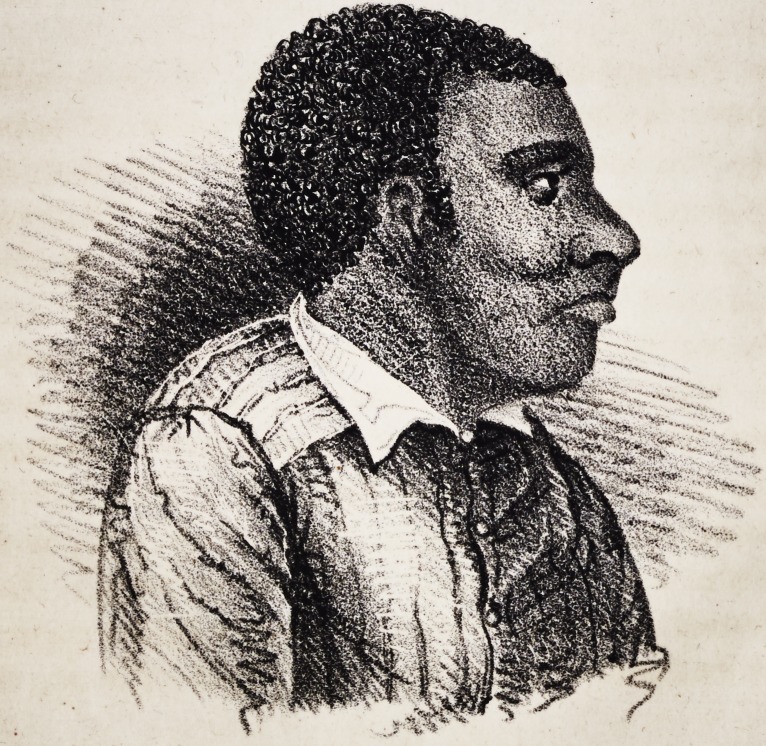# Osteo-Sarcoma of Lower Jaw—Amputation—Cure

**Published:** 1842-12

**Authors:** Charles Bell Gibson

**Affiliations:** Baltimore.


### ARTICLE IV.

*Osteo-Sarcoma of Lower Jaw—Amputation—Cure.* By CHARLES BELL GIBSON, M. D., of Baltimore.

MOSES LEE, blacksmith, fifty-one years of age, of robust make and active habits, a slave belonging to the estate of the late Richard O. Grayson, of Loudon county, Virginia, was placed in my hands in April, 1842, for the treatment of a tumour involving a large portion of the os maxillare inferius. About six years since, whilst engaged in ploughing, some obstacle occurring in the course of his furrow, caused the handle of his plough to be thrown violently upwards, striking him severely in the centre of the chin, and producing very severe pain in the part for a few days. In less than a week afterwards he discovered a slight swelling immediately over the spot, but as the pain had ceased, he paid little attention to it, unless when startled by a spontaneous jet of blood which occasionally occurred from between the first incisors of each side. The tumour increased slowly, at the end of a year having attained the size of a hickory-nut. From that period until I saw it, it increased more rapidly, but without pain; the only inconvenience being the difficulty of articulation and deglutition. The sketch fig. 1, gives a faithful representation of his appearance, previous to the operation. The tumour was confined to the lower jaw, having sprung apparently from the spot where the blow was received, directly in the middle of the chin, and extending itself with great regularity towards the angles of the bone. The limit of the disease was evidently just in front of the first molar tooth on each side, the measurement from one point to the other being exactly fourteen inches.

In an upward direction it extended nearly to the roof of the mouth, having pushed from their sockets all the incisors, the cuspid, and one of the bicuspids on both sides of the upper jaw, and separating the lips vertically to the extent of four and a half inches. Within the mouth and below, it extended along the under surface of the tongue to the anterior border of the sublingual gland, just in front of which, the uniformity of the surface was broken by a rising, or lobe, of the size of a black walnut, on which rested part of the under surface of the tongue. Over the whole surface of the tumour, which was intensely red, large veins were freely distributed, and at points in front were two deep ulcerations, from which, as well as from numerous small openings, was discharged a yellowish sero-purulent matter, small in quantity, and possessing but little odour. To the touch the general impression was hard, though in some places an elastic, and in others a decidedly fluctuating sensation was evident, especially in front, and where the tumour protruded between the lips. I performed the operation on the 12th of April, at 12 o'clock, in the presence of a number of the medical men of this city. I am particularly indebted to Drs. Baxley, T. Buckler and Theobald, for their valuable aid on the occasion.

The patient was seated in an ordinary chair, his head supported by an assistant. Standing in front, I made the first incision three-fourths of an inch to the left of the median line through the thickness of the lower lip in a straight line down as far as the diseased mass could be felt, an extent of about seven inches. The second incision was precisely similar, at the same distance from the median line on the right side, thus leaving attached to the tumour an inch and a half in width of lip and integuments, which, it was thought, would be superfluous in the union to be effected after the removal of the tumour.

A dissection was now rapidly made from the point of the first incision to the second molar tooth; the first molar on this side was now extracted by the dentist's key in order to remove with more facility the bone, just anterior to the second molar, and thus insure, as far as possible, the removal of all the diseased portion. The same course was followed on the right side. I now took a position directly behind the patient, his head resting on my breast, and the flap on the left side being held out of the way, sawed from above downwards through the bone on that side, cutting through the cavity occupied by the roots of the extracted molar. In the same way the right side of the bone was sawn through. Coming again in front and grasping the mass with the left hand, it was easily brought away by dividing its connections with the digastric, mylo-hyoid and genio-hyo-glossus muscles. The hemorrhage from the cavity thus exposed was profuse. The sub-mental and inferior coronary branches of the facial artery of both sides were easily secured, but the ranine and sublingual arteries gave us very considerable trouble, retiring out of sight, and from increasing weakness of the patient giving a scarcely perceptible jet. We removed him into the open air in a yard adjoining the room, and this with the aid of some wine and water, in about twenty minutes caused his pulse to rise, and the bleeding vessels were secured. From the division of the genio-hyo-glossus muscle, considerable inconvenience was produced in the disposition in the tongue to turn over and point down the pharynx.

A ligature passed through the frænum and secured, after the dressing of the wound was completed, to an iron wire contrivance, remedied the inconvenience. The apparatus consisted of an upright on either side of the head, having a horizontal position on a line with the mouth, and projecting some three inches beyond it. A single piece of iron wire was procured and bent for the purpose. Having carefully examined and determined the complete extirpation of the diseased bone, the edges of the wound were brought together and retained by hare-lip pins and the interrupted suture. Straps and roller completed the dressing, and the patient was put to bed. The oozing of blood continuing considerable, a piece of fine sponge was steeped in the muriated tincture of iron, and introduced into the mouth. The discharge was then entirely arrested.

Moses bore the operation extremely well. When put to bed his pulse was 60; feeble, but very even and regular. During the rest of the day he took occasionally a little very fluid barley water, finding considerable difficulty in swallowing. The evening and night he appeared comfortable, not complaining, and sleeping for the most part. Two medical students sat up with him through the night, and continued to do so as long as it was necessary.

*April* 13*th.* Find Moses doing extremely well; I have removed the sponge and wire apparatus, the disposition to swallow the tongue having ceased; pulse 62, regular; very little pain in the part. I have removed him to-day to a room in my own dwelling, where I can see him at any time more conveniently. He has taken to-day weak coffee and chicken water, swallowing readily by means of a catheter secured to a "sick cup." 3 P. M. Pulse 64, regular.

14*th.* About 7 o'clock last evening his pulse rose to 80, with-

For the American Journal of Dental Science out much heat of skin. The lips have become much swollen, with considerable heat; applied the sub. acet. plumbi. dilut., and opened his bowels by a simple enema; he slept well through the night; this morning at nine dressed the wound; it looks remarkably well, all below the lip having united by the first intention, except where the ligatures protrude; pulse 70; lip still much swollen. 3 o'clock, P. M. Moses has walked down stairs, and is now in the garden; same diet. 7 P. M. Pulse 74.

15*th*. Dressed the wound; very little discharge; lip continues much swelled and presents quite a sulcus above the first pin. He has been down stairs nearly all day walking about; spirits good; appetite famous.

16*th*. Passed a good night; swelling of lip subsiding; the upper pin came away to-day in dressing the wound. He has eaten to-day a large dish of mush besides his soup and barley water.

17*th*. After dinner to-day found Moses complaining a little of weakness; perhaps from over-exertion in walking about. Gave him some weak brandy toddy. 10 P. M. Stronger; pulse 70.

18*th*. Passed a very good night. Dressed the wound, and removed the last pin; two ligatures came away also. Diet to-day oysters and beer.

19*th*. Passed last night without having any one with him. Dressed the wound which has healed throughout, except at the upper part of the lip for about the eighth of an inch. This spot is improving under the nitrate of silver. The other ligatures were pulled away to-day.

26*th*. Moses has returned to Virginia to-day. Nothing worthy of notice has occurred since the last date. He has gone for the last three days without a bandage or strap of any kind. A thick line down the middle of his *chin* is the only evidence of his having submitted to an operation. There is very little deformity; the thick integuments most naturally representing the absent anterior portion of his maxilla inferior.

Fig. 2, represents the present appearance of Moses accurately. It only remains to be stated, that sections of the removed tumour made in various directions verified in all respects the description of the structure of osteo-sarcoma.

## Figures and Tables

**Figure f1:**
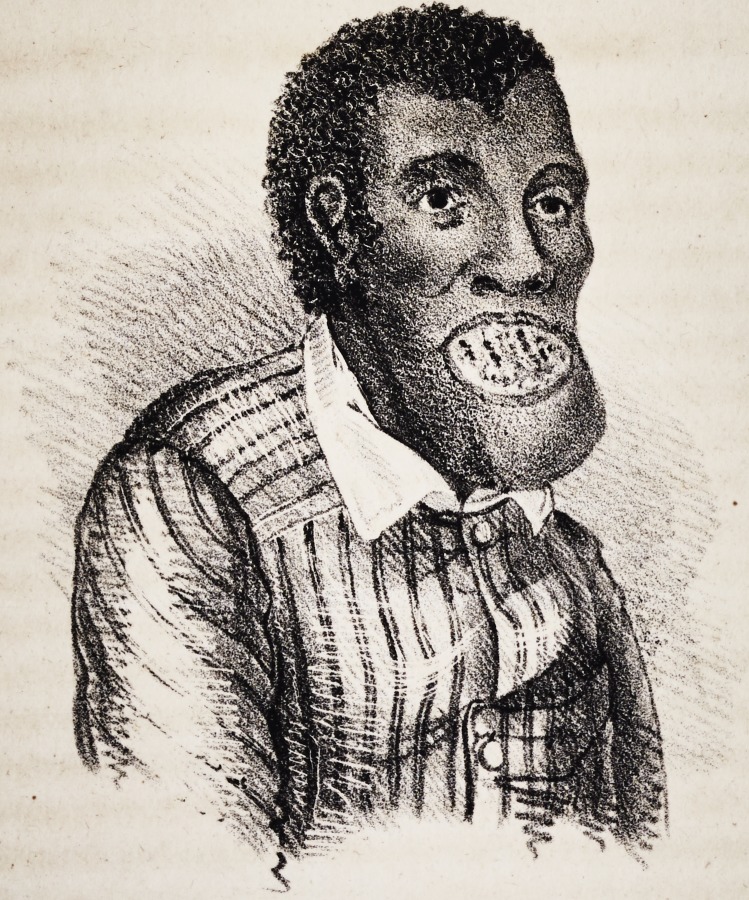


**Figure f2:**